# Anthocyanins in metabolites of purple corn

**DOI:** 10.3389/fpls.2023.1154535

**Published:** 2023-04-06

**Authors:** Taoyang Cai, Shangjie Ge-Zhang, Mingbo Song

**Affiliations:** ^1^ Aulin College, Northeast Forestry University, Harbin, China; ^2^ College of Science, Northeast Forestry University, Harbin, China; ^3^ College of Forestry, Northeast Forestry University, Harbin, China

**Keywords:** purple corn, anthocyanin, antioxidant activity (AA), gene regulation, mini-review

## Abstract

Purple corn (*Zea mays* L.) is a special variety of corn, rich in a large amount of anthocyanins and other functional phytochemicals, and has always ranked high in the economic benefits of the corn industry. However, most studies on the stability of agronomic traits and the interaction between genotype and environment in cereal crops focus on yield. In order to further study the accumulation and stability of special anthocyanins in the growth process of purple corn, this review starts with the elucidation of anthocyanins in purple corn, the biosynthesis process and the gene regulation mechanism behind them, points out the influence of anthocyanin metabolism on anthocyanin metabolism, and introduces the influence of environmental factors on anthocyanin accumulation in detail, so as to promote the multi-field production of purple corn, encourage the development of color corn industry and provide new opportunities for corn breeders and growers.

## 1 Introduction

Cereals are an important dietary resource for humans (Ciudad-Mulero et al., 2019; Guo et al., 2022). As one of the three major food grains in Asia, maize is the main source of food security and economic development in Sub-Saharan Africa, Latin America and the Caribbean (Grote et al., 2021; Soto-Gómez and Pérez-Rodríguez, 2022). With the increasing relentless pursuit of health in modern society, foods with high bioactive content have become popular. Purple corn stands out with its extremely high anthocyanin and phenolic compound content, and has attracted increasing attention (Lao et al., 2017; Colombo et al., 2021; Guo et al., 2021; Tiozon et al., 2022). As the main producer and exporter of purple corn in the world, Peru’s purple corn production accounts for about 23% of the total domestic corn production (Data of Ministry of Agriculture and Irrigation of Peru; Ritchie et al., 2022). On the one hand, purple corn has a wide range of industrial uses and can be used as photosensitizers for solar cells (Barba et al., 2022), natural colorants (Chatham et al., 2019; Cruzado et al., 2022), ethanol fuels (Somavat et al., 2018; Li et al., 2019; Ruan et al., 2019), etc. On the other hand, the antioxidant effect of anthocyanins attracted the attention of researchers earlier (Lieberman, 2007; Bendokas et al., 2020). Corn anthocyanins have antioxidant capacity and other biological effects. The anthocyanins in purple corn have a greater ability to scavenge free radicals than common antioxidants (Brewer, 2011), such as butylated hydroxyanisole (Felter et al., 2021), vitamin E (Blaner et al., 2021), catechin (Coșarcă et al., 2019) and quercetin wait (Xu et al., 2019). About 35.6-54.0% of the anthocyanins in purple corn are acylated, which has a positive effect on maintaining ***in vitro*** stability (Jing et al., 2007; McDougall et al., 2007), when the redox balance in the organism exceeds the capacity of the endogenous antioxidant defense system due to the excessive formation of free radical molecules, it can be used as a kind of exogenous antioxidant (Magaña Cerino et al., 2020). The antioxidant activity of phenolic compounds including anthocyanins increased with the maturity of purple corn, which was largely attributed to the change of its structure rather than its content (Hu and Xu, 2011; Harakotr et al., 2014). Therefore, anthocyanins in mature purple corn have rich nutritional and disease prevention value (Kang et al., 2012; Petroni et al., 2014; Gálvez Ranilla, 2020; Lee et al., 2020). Such as protecting cells (Hong et al., 2013; Poorahong et al., 2021), preventing cancer (Shi et al., 2021; Bars-Cortina et al., 2022; de Arruda Nascimento et al., 2022; Mottaghipisheh et al., 2022), preventing cardiovascular diseases (Wongsa, 2020; Dong et al., 2022; Miladiyah and Nuryadi, 2022) and improving eyesight (Ghosh and Konishi, 2007; Tandon, 2022).

To study the biological mechanism of special components in purple corn and provide new ideas for its cultivation and harvest has always been one of the research directions for scholars to promote the special crop industry (Escribano-Bailón et al., 2004; Zhang et al., 2019; Ranilla et al., 2021; Sunil and Shetty, 2022). Corn contains many secondary metabolites such as carotenoids and phenolic compounds (Acosta-Estrada et al., 2019; Tayal et al., 2020; Lee et al., 2021). Phenolic acids and flavonoids, as common phenolic compounds in corn kernels, exist in free, esterified (covalently bound with other molecules) and insoluble bound forms (Chen et al., 2021). As a member of the flavonoids family, anthocyanins are derived from the different degrees of hydroxylation and methoxylation of the flavin skeleton (ie, 2-phenylbenzopyran) (Ma et al., 2018; Alvarez-Suarez et al., 2021). Simple or acylated anthocyanins are mainly found in the aleurone layer of corn endosperm or pericarp and can greatly affect the color of the kernel (Pozo-Insfran et al., 2007; Žilić et al., 2016). Thapphasaraphong et al. (2016) found that cyanidin-3-glucoside is the most important anthocyanin component in grain by thin layer chromatography analysis. In addition, due to the high content of functional pigments in corn in inedible husks, cobs and silks, for example, the anthocyanin content in corn husks is between 17.3% and 18.9% of the dry weight, which is about 10 times the current standard purple corn kernel content of 1.78%, the by-products of purple corn have also been selected as potential sources for extracting anthocyanins (Li et al., 2008; Yang et al., 2008; Deineka et al., 2016; Chaiittianan et al., 2017). The anthocyanins in different tissues of different types of purple corn are shown in **Table 1**.

**Table 1:** Anthocyanin content in different tissues of different types of purple maize.

Source	Tissue	Main Anthocyanin Species	Anthocyanin Content (mg/100g Dry Weight)	Ref.
Peru	Cob	Cyanidin-3-glucoside, Pelargonidin-3-glucoside, Peonidin-3-glucoside	2600-3800	(de Pascual-Teresa et al., 2002; Monroy et al., 2016)
Bolivia	Kernels	Cyanidin 3-β-glucoside	\	(Nakatani et al., 1979)
Andes	Total	Cyanidin-3-glucoside, Pelargonidin-3-glucoside, Peonidin-3-glucoside	1642	(Cevallos-Casals and Cisneros-Zevallos, 2003; Pedreschi and Cisneros-Zevallos, 2007)
\	Bran	Cyanidin-3-O-glucoside, Cyanidin-3-O-(6-malonylglucoside)	36.25	(Chen et al., 2018)
Mexico	Husk	Cyanidin-3-glucoside, Pelargonidin-3-glucoside, Peonidin-3-glucoside, Pelargonidin-3-(6’’-malonylglucoside), Cyanidin-3-(6’’-malonylglucoside), Peonidin-(6’’-malonylglucoside)	2432-2580	(Fernandez-Aulis et al., 2019)
China	Cob	Cyanidin-3-glucoside, Pelargonidin-3-glucoside, Peonidin-3-glucoside, Pelargonidin-3-(6’’-malonylglucoside), Cyanidin-3-(6’’-malonylglucoside), Peonidin-(6’’-malonylglucoside)	185.1	(Yang and Zhai, 2010a)
China	Kernels	Cyanidin-3-glucoside, Pelargonidin-3-glucoside,Peonidin-3-glucoside	55.8-304.5	(Zhao et al., 2009; Yang and Zhai, 2010b)
Thailand	Kernels	Cyanidin-3-glucoside	1970	(Harakotr et al., 2014)

In addition, the anthocyanin composition and total phenolic content of purple corn samples under different planting conditions were highly variable, the monomeric anthocyanins content ranged from 290 to 1333 mg/100g cyanidin 3-glucoside equivalents of drymatter, while the total phenolic content ranged from 950 to 3516 mg/100g of dry matter as gallic acid equivalents (Jing et al., 2007). This is due to the fact that various factors can affect the accumulation and stability of anthocyanins, including genetics (Coe, 1994; Khampas et al., 2015; Peniche-Paviía and Tiessen, 2020), agronomy (Nurnawati, 2020), pH value used for extraction (Qin et al., 2019; Rodriguez-Amaya, 2019; Vidana Gamage et al., 2022), temperature (Zhao et al., 2008; Lao and Giusti, 2017; Gullón et al., 2020) and light intensity (Chalker-Scott, 1999; Vidana Gamage et al., 2022), which will be specifically mentioned in the second section. At present, the methods for extracting total anthocyanins and total phenolic compounds in purple corn dry core mainly include ultrasonic-assisted extraction (Chen et al., 2018; Muangrat et al., 2018; Xue et al., 2021), microwave-assisted extraction (Yang and Zhai, 2010a; Herrman et al., 2020; Jayaprakash et al., 2022), and organic solvent extraction (Lao and Giusti, 2018). Usually, high performance liquid chromatography and spectrophotometry are used for identification and analysis (Wu et al., 2006; Singh et al., 2020).

This mini-review introduces the various values of purple corn that are inseparable from the content of anthocyanins. In the second section, the basic biological mechanism of the synthesis of anthocyanins and other substances in purple corn will be described, and the pH, light and The influence of temperature (Section III), at the end of the review, a summary and outlook on how to make full use of anthocyanins in purple corn and improve their recovery and quality.

## 2 Synthesis mechanism

As a kind of water-soluble natural pigment widely present in plants in nature, anthocyanins endow many plants with bright and attractive colors and are valuable sources of bioactive compounds. However, the lack of genomic data on the regulatory mechanism of anthocyanin biosynthesis in purple maize (*Zea Mays* L.) has hindered the selection process of purple maize varieties. With the development of molecular biology and bioinformatics, a large number of studies have revealed the complexity of the molecular regulation mechanism of the anthocyanin synthesis pathway and its huge differences among different plants. Among them, structural genes and regulatory genes determine the synthesis and regulation of anthocyanins in purple maize.

### 2.1 Regulation of anthocyanin biosynthesis

#### 2.1.1 Regulatory genes

The biosynthetic pathway of anthocyanins has been described in Arabidopsis (Solfanelli et al., 2006; Cappellini et al., 2021), tomato (Butelli et al., 2008; Wang et al., 2020), rice (Mackon et al., 2021; Xia et al., 2021) and many other species (Chen et al., 2012; Feng et al., 2018), mostly through the interaction of regulatory genes and plant hormones (Hao et al., 2021; Paulsmeyer and Juvik, 2022). With the discovery of potential key regulatory genes, the biosynthetic pathway of anthocyanins in purple maize has also been well established (Zhang et al., 2020; Banerjee et al., 2022). Anthocyanin biosynthesis genes are mainly regulated by several families of transcription factors (TFs) at the mRNA level (Zhang et al., 2016), that is, anthocyanins are regulated at the transcriptional level by the MYB-bHLH-WDR (MBW) complex (Lloyd et al., 2017; Sun et al., 2022), and the regulatory genes of the complex They are MYB (V-myb myeloblastosis viral oncogene homolog), WDR (WD-repeat) and bHLH (Basic helix-loop-helix) (Sharma et al., 2011). The distribution of purple maize anthocyanins in different tissues is determined by the tissue-specific expression of regulatory genes. Booster1 (*B1*) and Plant color1 (*Pl1*) are the bHLH and MYB regulatory factors, respectively, most often associated with regulation in plant tissues (Styles and Coe, 1986; Coe et al., 1988; Chatham and Juvik, 2021). A recessive intensifier of anthocyanin biosynthesis in maize, *in1* (intensifier1), encodes a bHLH type protein with high sequence similarity to *R1* and *B1* (Burr et al., 1996; Cone, 2007; Chatham et al., 2019), certain alleles of *R1* operate in pericarp and certain *B1* alleles operate in aleurone (Portwood et al., 2019). In brief, the interaction of these transcription factors with their target genes leads to the spatiotemporal biosynthesis of maize anthocyanins (He et al., 2021). Moreover, the researchers used the Agrobacterium-mediated method to transfer the combination of *ZmC1* and *ZmR* belonging to the MYB-type and bHLH families in maize to wheat, and overexpressed anthocyanin-rich germplasm wheat (Riaz et al., 2019), indicating that transcription modulation of factor expression was effective in increasing anthocyanin content (Jian et al., 2019).

#### 2.1.2 Structural genes

Transcriptional regulators not only determine the spatial and temporal patterns of anthocyanin accumulation, but also activate the expression of anthocyanin structural genes (Gordeeva et al., 2019; Khusnutdinov et al., 2021; Yan et al., 2021). The expression of structural genes in high anthocyanin tissues of purple maize was always higher than that in low anthocyanin tissues (Kaur and Singh, 2022). Structural genes directly encode enzymes required in the anthocyanin biosynthetic pathway, such as Phenylalanine ammonia lyase, Chalcone synthase, Chalcone isomerase, Flavanone 3-hydroxylase, Flavonoid 3’- hydroxylase, Dihydroflavonol-4-reductase, Leucoanthocyanidin dioxygenase, Anthocyanidin 3-O-glucosyltransferase, etc (Li et al., 2020; Liu et al., 2021; Kaur et al., 2022). Through transcriptome sequencing, researchers found that anthocyanin biosynthesis is mainly regulated by structural genes CHS, CHI, F3H, DFR, LODX and GST, among which CHS is an early biosynthesis gene of anthocyanin (Wang et al., 2022). 72% of the structural genes regulating anthocyanin synthesis were up-regulated, and most of the differentially expressed genes had the highest expression level at 34 day after pollution, when the ratio of anthocyanin content to fresh weight was also the highest (Ming et al., 2021). Indeed, the carbon flux to anthocyanins ***via*** the flavonoid pathway in purple maize is complex (Chatham and Juvik, 2020).

### 2.2 Steps of anthocyanin biosynthesis

**Figure 1:**
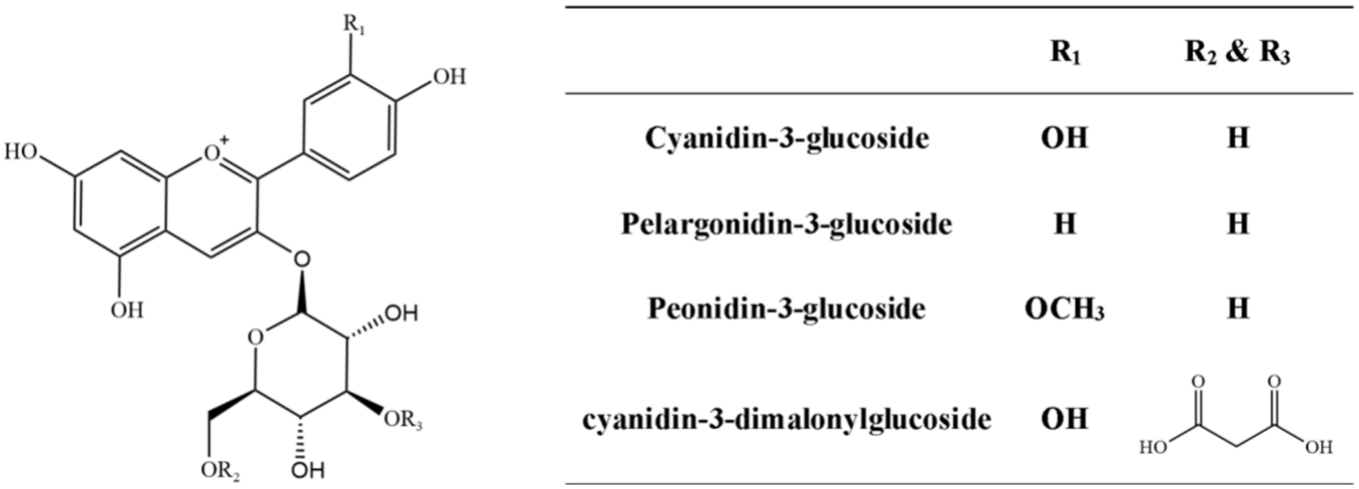
Examples of major anthocyanin species in Andean maize.

Chemically, anthocyandins are polyhydroxy/polymethoxy glycosides derived from anthocyanins (Holton and Cornish, 1995). The Andes region of South America is the birthplace of purple corn, the anthocyanins present in Andean purple corn, flowers, leaves, cobs, and kernels have previously been characterized, and the major anthocyanins found were cyanidin-3-dimalonylglucoside, cyanidin-3-glucoside, pelargonidin-3-glucoside, peonidin-3-glucoside, and their respective malonated counterparts (Fossen et al., 2001; Aoki et al., 2002; Hong et al., 2020). **Figure 1** shows the major anthocyanin species in the most representative Andean purple corn.

**Figure 2:**
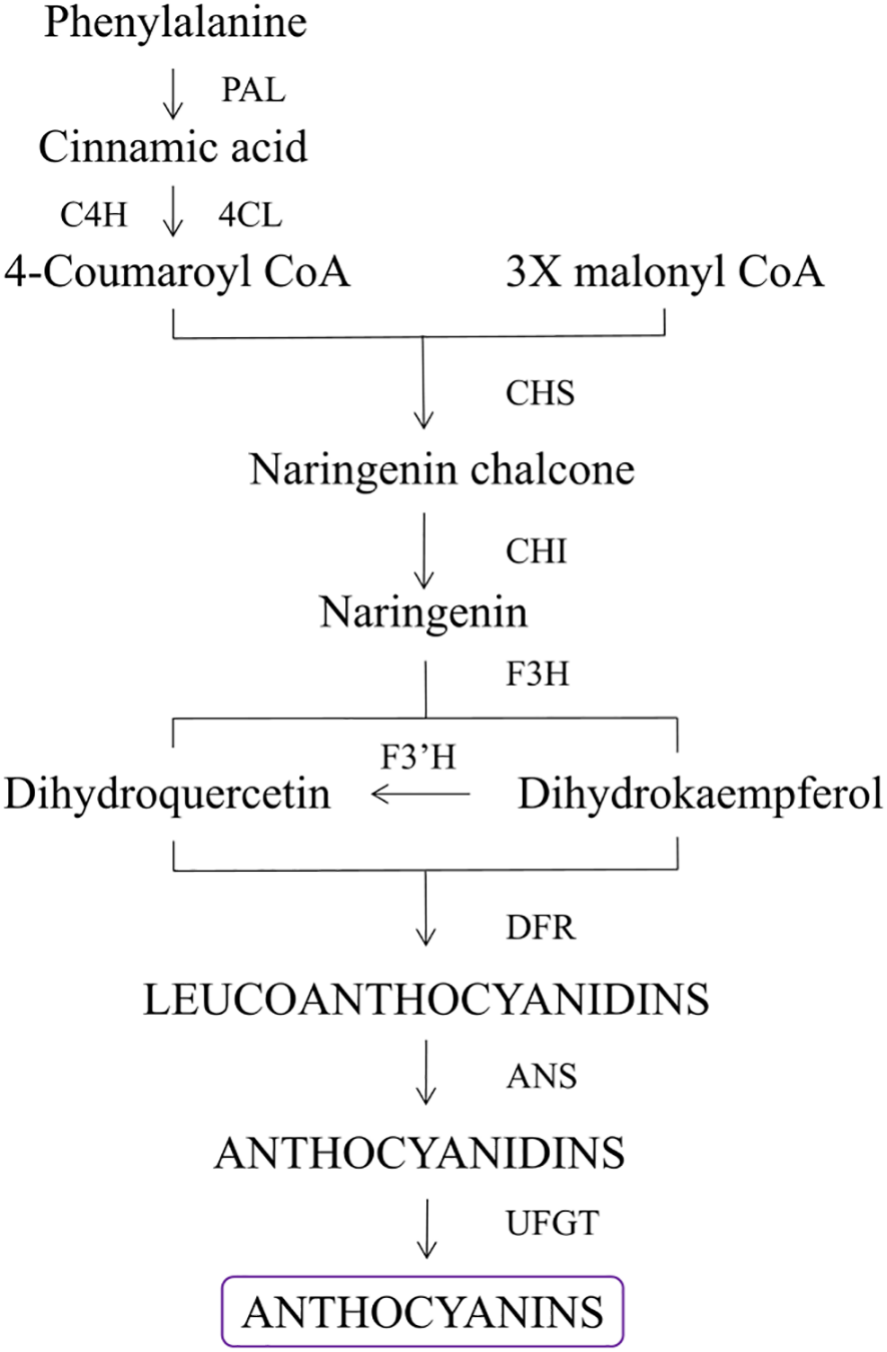
Synthetic pathway of simplified version of anthocyanins.

The production of flavonoids including anthocyanins can be briefly described as the following steps (**Figure 2**). In purple corn, the synthesis of anthocyanins originates from phenylalanine. First, phenylalanine ammonia lyase (PAL) deaminates phenylalanine into cinnamic acid, which is then converted to the main precursor of anthocyanins, 4-coumaroyl CoA (Ayvaz Sönmez et al., 2021). One 4-coumaroyl CoA and three malonyl CoA molecules can be condensed under the action of Chalcone synthase (CHS) to generate naringin chalcone, which is an early key reaction in the biosynthesis of flavonoids and is generally considered to be the rate-limiting step of this pathway step (Dixon et al., 2002). Chalcone isomerase (CHI) isomerizes naringenin chalcone to colorless naringenin. Catalyzed by flavanone 3-hydroxylase (F3H), naringenin is hydroxylated at the third position to generate dihydrokaempferol (DHK). Next, A flavanoid 3’-hydroxylase (F3’H) can use either naringenin or DHK as substrates, adding a hydroxyl group to the 3’position of dihydroflavonols to create dihydroquercetin (DHQ). Dihydroflavanols, DHQ, and DHK are reduced to colorless Leucoanthocyanidins by Dihydroflavonol-4-reductase (DFR). Futher, Leucoanthocyanidins serve as substrates for anthocyanidin synthase (ANS) to make anthocyanidins. Finally, the colorful anthocyanindins are then catalyzed by flavonoid-3-O-glucosyltransferase (UFGT) for glycosylation and form morestable molecules, anthocyanins (He et al., 2010). The synthesized anthocyanins will be transported into the vacuoles by transporters and stored in the form of colored aggregates, called anthocyanin vacuolar inclusions (Goodman et al., 2004; Lago et al., 2013).

## 3 Environmental influencing factors

In addition to the genetic determination of purple maize itself, environmental factors including ultraviolet radiation, temperature and water stress have been shown to induce the accumulation of anthocyanins in plants (Straus, 1959; Chalker-Scott, 1999; Steyn et al., 2002; Ayala-Meza et al., 2023). In fact, in purple maize, environment accounted for the largest portion (77.83%) of the total variation in grain yield (MITROVIÃ et al., 2012). In addition, the environmental factors selected during extraction will also have an impact on the final anthocyanin content obtained in the industry, because anthocyanin is more stable under acidic and low temperature conditions.

### 3.1 Soil

The soil environment can significantly affect the accumulation of anthocyanins, such as the application of nitrogen fertilizers (Sugaya et al., 2001; Utasee et al., 2022). Mollah et al. (2020) applied nitrogen, phosphorus and potassium fertilizers (3.05 tons ha^-1^) and humic acid (20 kg ha^-1^) to the soil to increase the soil pH and increase the cation exchange capacity to 25.8 CmoL(+)/kg, which had a significant effect on the growth and production parameters of purple maize. Jing et al. (2007) found that different concentrations or forms of potassium salts had no significant effect on the anthocyanin content of purple corn cobs. Metal ions affect the accumulation of anthocyanins. Janeeshma et al. (2021) found that the accumulation of anthocyanins in maize plant leaves increased with the increase of soil element zinc content. Trace metal ions absorbed from soil usually accumulate in vacuoles and form stable complexes with anthocyanins, thereby affecting their color and increasing their stability (Sigurdson, 2016; Enaru et al., 2021). In addition, silicon treatment can enhance the drought tolerance of purple maize, which also has beneficial effects under abundant water conditions (Goto and Kondo, 1991; Özdemir, 2021).

### 3.2 Temperature

Temperature will also affect the accumulation of anthocyanins in purple corn. The low temperature induced the expression of regulatory and structural genes such as MYB10 and bHLH3/33, and the transcription of anthocyanin-related synthetases in maize seedling sheaths. The level remained stable at low temperature (10°C) and then rose rapidly, and dropped to the pretreatment level within 2 days after the cold-stressed seedlings returned to normal temperature (25°C) (Christie et al., 1994). At normal temperature, Paucar-Menacho et al. (2017) used response surface analysis found that the concentration of anthocyanins in purple maize sprouts increased with the extension of germination time at 26°C within 63 h. Vilcacundo et al. (2020) found that the Andean purple corn had the highest germination rate of 63.33% at 25°C, and the germination rate decreased with the increase of germination temperature. The germination rate was between 9.33% and 26.00% at 40°C. High temperature (32°C) induced the expression of MYB16, resulting in a “residue” effect, lower synthesis and accumulation of anthocyanins in grains and ears (Wang et al., 2016; Aguilar-Hernández et al., 2019). Also, at higher temperatures, due to enhanced superoxide dismutase activity and increased malondialdehyde content, anthocyanins will degrade due to increased H_2_O_2_ concentration (Yüzbaşıoğlu et al., 2017; Bayat et al., 2018).

### 3.3 Illuminance

The influence of temperature and light on the growth and metabolism of purple corn is inseparable. Janda et al. (1996) transferred corn seedlings treated with low temperature and dark to normal temperature and light, and found that the content of plant pigment increased threefold in one day. Independently, light is also an important factor controlling anthocyanin synthesis (Mancinelli, 1983; Byrnes, 2011; Pech et al., 2022). Anthocyanin synthesis and accumulation in purple maize seedlings are the result of lightinduction (Gu et al., 2018; Shimakawa and Miyake, 2021). The *Lc* (leaf color) gene is an anthocyanin-regulated gene of bHLH (basic/helix-loophelix) in maize. Under strong light conditions, the LC transcription factor promotes and induces the production of anthocyanins in vegetative and reproductive tissues (Fan et al., 2016). Light is essential for the induction of PAL and CHS and the accumulation of anthocyanins, and the accumulation of CHS and PAL mRNA is controlled by three photoreceptors: UV B (Ultraviolet Radiation B) receptor, blue light receptor and phytochrome (Alokam et al., 2002). It is worth noting that the light absorption of anthocyanins is not only attributed to the overall ring structure and conjugated double bonds, but also depends on the light quality, luminous flux, and exposure time. Therefore, lighting conditions need to be optimized for their intensity, exposure time and type (Pech et al., 2022). Guo et al. (2008) found that too much radiation from UV B may inhibit anthocyanin synthesis through DNA damage.

### 3.4 Extraction

**Figure 3:**
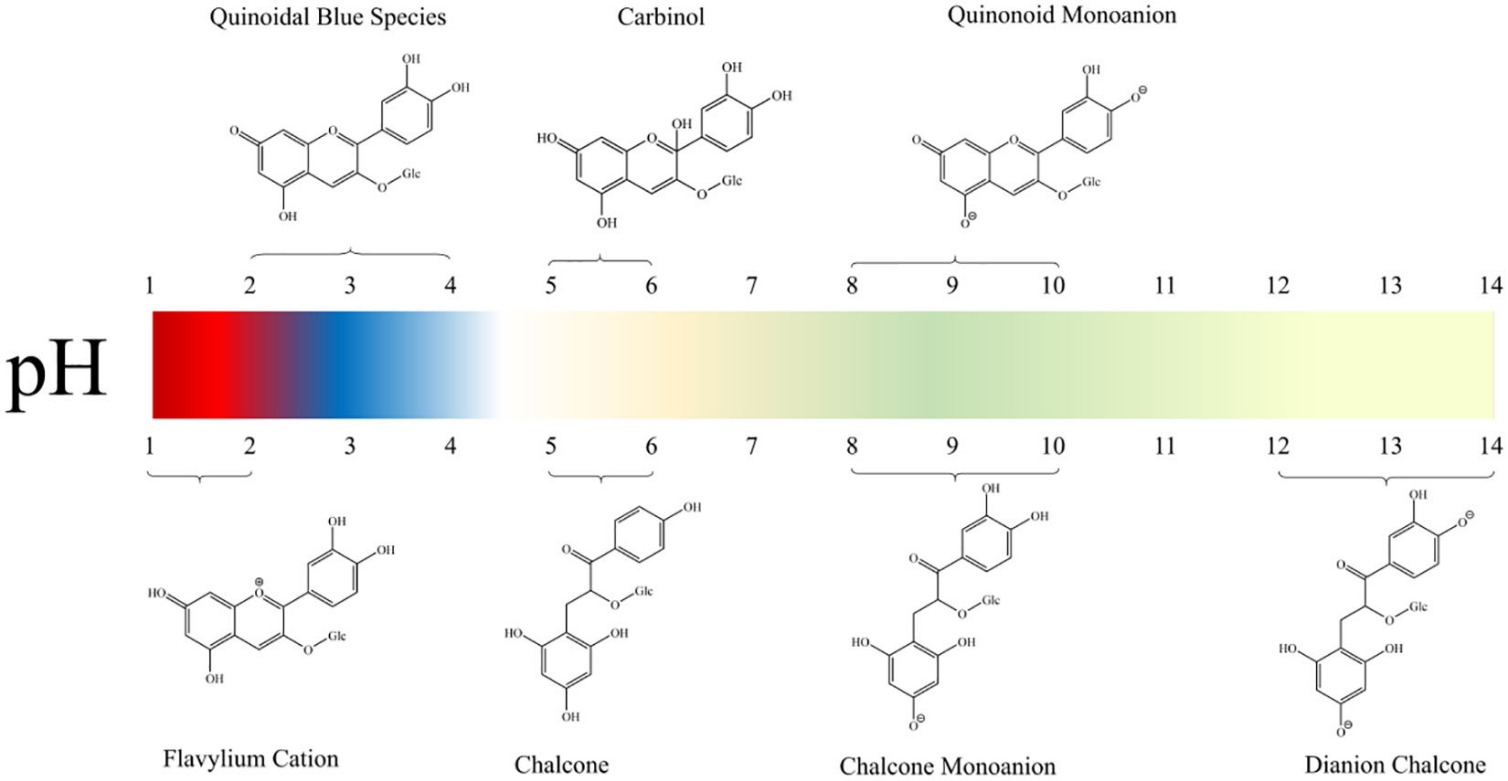
Color of anthocyanins at different pH value.

In the extraction of anthocyanins, anthocyanins in purple corn are often in an equilibrium state between the colored cation form and the colorless half ketone formed by hydration, which is directly affected by pH (**Figure 3**). With the change of pH, anthocyanins undergo stability changes and reversible structural changes in different water environments, so the color also changes drastically (Vankar and Srivastava, 2010).

Anthocyanins have the highest color stability at lower pH and are less stable at neutral or alkaline pH (Amogne et al., 2020). When the pH value is around 1, anthocyanins are protonated and mainly exist in the form of flavin cations, which are easily soluble in water and turn red (Cooper-Driver, 2001; Harborne, 2013). The quinoidal blue species is abundantly produced at pH value from 2 to 4 (Basílio et al., 2021). When the pH increased to 4-6, the flavin cation was rapidly hydrolyzed at the 2-position under the nucleophilic attack of water to produce a colorless carbinol pseudoradical and a pale yellow chalcone (Kallam et al., 2017). Around pH 8-10, further deprotonation, shifting the color of medium to green, when the ionized chalcone and ionized quinoid (Levi et al., 2004). At pH values greater than 12, dianion chalcone is the major compound, producing a yellow color in the solution (Brouillard and Delaporte, 1977; Petrov et al., 2013).

Heat-induced color changes are permanent and irreversible (Burkinshaw and Towns, 1998; Halász et al., 2023). Anthocyanins stored in acylated form are more stable at different temperatures than non-acylated anthocyanins (Leonarski et al., 2022; Luo et al., 2022). Yang et al. (2009) used ethanol to extract anthocyanins from purple corn and found that the yield was higher at 10°C to 50°C. After dissolving the purple corn flour extract, Aprodu et al. (2020) determined according to the pH difference method that anthocyanins can still maintain a certain stability at 80°C to 120°C. However, too high temperature will lead to the thermal degradation of anthocyanins and the decline of productivity in the production process (Mercadante and Bobbio, 2008).

## 4 Summary and outlook

Anthocyanins, the multifunctional active substances in purple corn, may be of interest to various industries such as dietary supplements, food additives, and cosmetics. This paper briefly introduces the anthocyanin content in purple corn from different sources, focuses on the metabolic pathway of anthocyanin and the regulatory genes behind it and the structural genes encoding enzymes, and explains the impact of environmental factors on the growth process and extraction of purple corn. In view of the current hot issues related to the research on anthocyanins and phenolic compounds in purple corn, we propose the following outlook:

(1) Due to the high content of functional pigments in by-products such as kernel, cob, and silk, it is urgent to improve the utilization of purple corn. Moreover, if more by-products of purple corn are developed, not just anthocyanins, purple corn may generate additional value in the future.
(2) The effects of anthocyanins on purple waxy corn have been studied, such as variety, environment and their interaction. Advances in functional genomic analysis of anthocyanin biosynthetic pathways using recombinant DNA technology and the combination of plant metabolic engineering with biotechnological tools will be a promising strategy to increase anthocyanin production.
(3) Since traditional breeding methods are relatively limited by the phenotypic cost and yield of nutritional traits, molecular marker-assisted selection methods are particularly useful for improving nutritional traits, and precise positioning must be combined with traditional methods to improve useful phytochemicals to develop Healthier and higher quality breeding lines.
(4) At present, there are few studies on how soil pH affects anthocyanin accumulation during purple corn cultivation, and most of them focus on the pH analysis of anthocyanin extraction from purple corn. Moreover, there is a browning effect in anthocyanin extracts, which is often accompanied by a decrease in the concentration of anthocyanins, which affects the extraction yield. How to better avoid the browning effect of anthocyanins in purple corn is also an urgent problem to be solved.

## Author contributions

TC: Writing – original draft, Writing – review & editing, Investigation, Visualization, Methodology; SG-Z: Writing – original draft, Writing – review & editing, Investigation; MS: Resources; Supervision, Writing – review & editing. All authors contributed to the article and approved the submitted version. 
